# Sub-Toxic Human Amylin Fragment Concentrations Promote the Survival and Proliferation of SH-SY5Y Cells via the Release of VEGF and HspB5 from Endothelial RBE4 Cells

**DOI:** 10.3390/ijms19113659

**Published:** 2018-11-20

**Authors:** Giuseppe Caruso, Claudia G. Fresta, Giacomo Lazzarino, Donatella A. Distefano, Paolo Parlascino, Susan M. Lunte, Giuseppe Lazzarino, Filippo Caraci

**Affiliations:** 1 Oasi Research Institute—IRCCS, 94018 Troina, Italy; carafil@hotmail.com; 2 Ralph N. Adams Institute for Bioanalytical Chemistry, University of Kansas, Lawrence, KS 66045, USA; forclaudiafresta@gmail.com (C.G.F.); slunte@ku.edu (S.M.L.); 3 Department of Pharmaceutical Chemistry, University of Kansas, Lawrence, KS 66045, USA; 4 Institute of Biochemistry and Clinical Biochemistry, Catholic University of the Sacred Heart, 00168 Rome, Italy; g.lazzarino@libero.it; 5 Fondazione Policlinico Universitario A. Gemelli IRCCS, 00168 Rome, Italy; 6 Department of Biomedical and Biotechnological Sciences, Division of Medical Biochemistry, University of Catania, 95125 Catania, Italy; donadist@hotmail.it (D.A.D.); paolo.parlascino22@gmail.com (P.P.); 7 Department of Chemistry, University of Kansas, Lawrence, KS 66045, USA; 8 Department of Drug Sciences, University of Catania, 95125 Catania, Italy

**Keywords:** amylin, β-amyloid, Alzheimer’s disease, vascular endothelial growth factor, HspB5, neuroprotection

## Abstract

Human amylin is a 37-residue peptide hormone (hA1-37) secreted by β-cells of the pancreas and, along with insulin, is directly associated with type 2 diabetes mellitus (T2DM). Amyloid deposits within the islets of the pancreas represent a hallmark of T2DM. Additionally, amylin aggregates have been found in blood vessels and/or brain of patients with Alzheimer’s disease, alone or co-deposited with β-amyloid. The purpose of this study was to investigate the neuroprotective potential of human amylin in the context of endothelial-neuronal “cross-talk”. We initially performed dose-response experiments to examine cellular toxicity (quantified by the [3-(4,5-dimethylthiazol-2-yl)-2,5-diphenyltetrazolium bromide] MTT assay) of different hA17–29 concentrations in endothelial cells (RBE4). In the culture medium of these cells, we also measured heat shock protein B5 (HspB5) levels by ELISA, finding that even a sub-toxic concentration of hA17–29 (3 µM) produced an increase of HspB5. Using a cell medium of untreated and RBE4 challenged for 48 h with a sub-toxic concentration of hA17–29, we determined the potential beneficial effect of their addition to the medium of neuroblastoma SH-SY5Y cells. These cells were subsequently incubated for 48 h with a toxic concentration of hA17–29 (20 µM). We found a complete inhibition of hA17–29 toxicity, potentially related to the presence in the conditioned medium not only of HspB5, but also of vascular endothelial growth factor (VEGF). Pre-treating SH-SY5Y cells with the anti-Flk1 antibody, blocking the VEGF receptor 2 (VEGFR2), significantly decreased the protective effects of the conditioned RBE4 medium. These data, obtained by indirectly measuring VEGF activity, were strongly corroborated by the direct measurement of VEGF levels in conditioned RBE4 media as detected by ELISA. Altogether, these findings highlighted a novel role of sub-toxic concentrations of human amylin in promoting the secretion of proteic factors by endothelial cells (HspB5 and VEGF) that support the survival and proliferation of neuron-like cells.

## 1. Introduction

Human amylin (hA) is a peptide hormone composed of 37 amino acids synthesized in β-cells of the pancreas implicated in the regulation of glucose metabolism [1] and co-secreted with insulin in a 1:20 ratio [2]. In vivo, the “final” full-length peptide (hA1-37) is obtained after the cleavage of its precursor of 89 amino acids in a multi-step process [3,4]. The first 22 amino acids, corresponding to the signal sequence, are released during the first processing step, while the remaining 67 amino acids (the pro-hormone form of hA) are converted into the final peptide after the hydrolysis of both N- and C-terminal regions by the prohormone convertases 2 (PC2) and PC1/3 [3]. The soluble monomer of hA can undergo aggregation through a step-by-step process, leading to the formation of higher molecular weight multimers with toxic properties [5]. Deposition of hA aggregates has been found in about 90% of patients suffering from type 2 diabetes mellitus (T2DM) [6] and it plays a central role in β-cells death [7]. The same hA deposits, alone or in co-presence of β-amyloid (Aβ), were found in the brain of both Alzheimer’s disease (AD) and T2DM patients [8], showing once again that hA and Aβ represent key factors with common linking pathobiological mechanisms both in AD and TD2M [9].

In the last two decades, cell culture medium conditioned by different cell types has been used extensively to study the cellular cross-talk in vitro, with particular attention to the role exerted by secretory molecules. Conditioned medium (CM) collected from pure cultures of cortical astrocytes protected neurons against Aβ toxicity via a paracrine signaling mediated by the secreted transforming growth factor beta 1 [10]. In a different study, Polazzi and Contestabile [11] showed the ability of CMfrom mature neurons to maintain a controlled inflammatory state through the production of factors inducing activated microglia cell death. Interestingly, their results also suggested that the medium collected from immature neurons may favor, on the contrary, the survival of microglia during the development process. Moreover, it has been shown that cerebral endothelial-conditioned media promote survival and proliferation of oligodendrocyte precursor cells via Akt and Src signaling pathways [12].

Heat shock protein B5 (HspB5)(also known as αB-crystallin) is a chaperone belonging to the small heat shock protein family (HspBs). The molecular chaperone activity of HspBs is principally involved in the recognition and refolding of proteins in different unfolded states [13]. A high expression of crystallins is a result of cellular stress response, since HspB4 and HspB5 increase cellular resistance to stress-inducible apoptosis [14,15,16].

Vascular endothelial growth factor (VEGF) is an endothelial cell-specific mitogen, which is able to increase vascular permeability and angiogenesis [17]. Endothelial cells are known to produce a wide range of trophic factors involved in neuronal homeostasis, including a large amount of VEGF in vitro and in vivo [18,19,20]. The biological activity and the neuroprotective effect of VEGF have been investigated in neuronal cultures, showing its ability in enhancing neuronal proliferation and preventing Aβ-induced neurotoxicity [21].

It has been demonstrated that VEGF expression is post-transcriptionally regulated by HspB5 in retinal pigment epithelium cells subjected to hypoxia. A decrease in oxygen availability dramatically reduces phosphorylated HspB5, thus negatively affecting VEGF expression, and finally leading to imperfect vascularization and increased endothelial apoptosis [22]. The direct interaction of HspB5 with VEGF has been shown to protect this protein against unfolding and aggregation [13]. Additionally, van de Schootbrugge et al. [23] demonstrated that HspB5 expression enhances VEGF secretion, while Kerr and Byzova [24] have shown that phosphorylated HspB5 chaperones misfolded and monoubiquitinylated VEGF (produced under hypoxic stress) to the endoplasmic reticulum, thereby preventing its accumulation in the cytoplasm and avoiding further stressing conditions to the cell.

The present study examines the potential protective effects exerted by human amylin on neuronal-like cells through the secretion of proteic factors by endothelial cells. As a first step, we examined the time course of hA17–29 β-amyloid fibril formation and then tested the toxicity of hA aggregates in endothelial cells. Among the various types of hA fragments capable of forming amyloids in vitro, we focused our attention on the activity of hA17–29 that, during the last decade, has been proposed as a useful tool able to mimick some of the key biological activities of hA1-37 [25,26,27]. By using a previously established experimental model (RBE4–SH-SY5Y) to study the interaction between endothelial and neuronal-like cells [28,29], we investigated the ability of hA17–29 in inducing the release of HspB5 and VEGF by RBE4 cells, as well as the effect of RBE4 CMon neuroblastoma SH-SY5Y cells challenged with hA17–29. In the present paper, we provided evidence that sub-toxic concentrations of hA17–29 enhance the release of proteic factors from RBE4, finally decreasing hA17–29-induced toxicity in SH-SY5Y cells via a receptor-mediated mechanism.

## 2. Results

### 2.1. Time Course of In Vitro hA17–29 Aggregation

**Figure 1:**
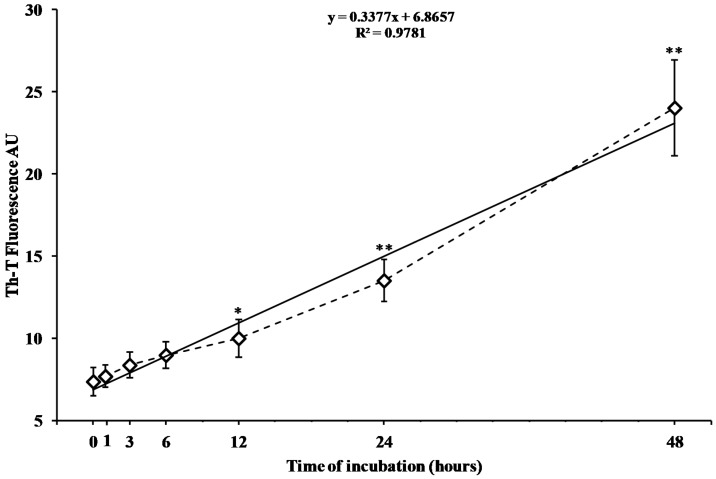
Change in the fluorescence intensity of the Th-T assay caused by the aggregation of the peptide fragment hA17–29. The peptide fragment hA17–29 (100 µM) was incubated in 0.01 M phosphate buffered saline (PBS), pH 7.4, at 37 °C for different times (0, 1, 3, 6, 12, 24, and 48 h). Fluorescence was monitored as a function of time in a 96-well plate using a microplate reader (LabSystems-Multiskan Ascent 354 Microplate Reader, San Diego, CA, USA). The fluorescence intensity was measured at 450 nm wavelength excitation/482 nm wavelength emission after 10 min of incubation of Th-T (3 µM) with the peptide fragment. The final fluorescence values were calculated by subtracting the fluorescence produced by control solutions (Th-T alone and aggregates in solution in absence of Th-T). Data are the mean of five independent experiments (an average of six readings was considered for each sample). Standard deviations are represented by vertical bars. The dotted line is the trend of the experimental points (rhombus); the solid line is the best fitting straight line. * Significantly different from 0 time, *p* < 0.01; ** significantly different from 0 time, *p* < 0.001.

Figure 1 reports the time course changes in aggregate formation of freshly prepared hA17–29 fragment after 0, 1, 3, 6, 12, 24, and 48 h of incubation at 37 °C, as evaluated by the change in fluorescence intensity when using the thioflavin T (Th-T) assay.

Fluorescence increased linearly as a function of the incubation time. Changes in fluorescence were not significant during the first four incubation times (0, 1, 3, and 6) analyzed (Figure 1). Fluorescence intensity significantly increased after 12, 24, and 48 h of incubation (+35%, +83%, and +226%, respectively, *p* < 0.01 compared to the value at zero time), indicating a quite long lag time before the aggregation process took place, as well as a relatively slow rate in the phenomenon of hA17–29 aggregation.

### 2.2. Effect of hA17–29 Fragment on RBE4 Cell Viability and Release in the Medium of Potentially Protective Proteins

**Figure 2:**
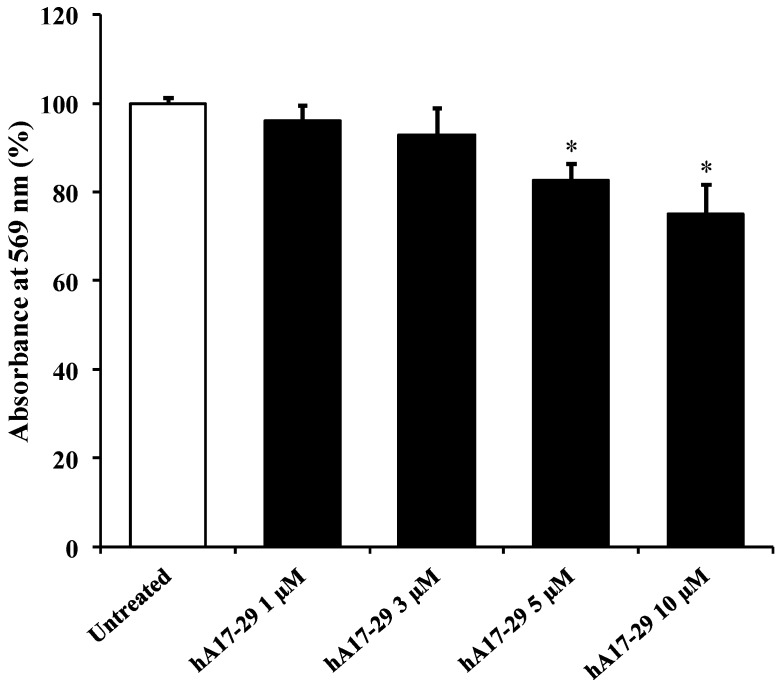
Change in the cell viability caused by challenging for 48 h endothelial cells (RBE4) cells with different concentrations (1, 3, 5, and 10 µM) of freshly prepared hA17–29 peptide fragment. Cell viability was determined using the [3-(4,5-dimethylthiazol-2-yl)-2,5-diphenyltetrazolium bromide] MTT assay. MTT solution (1 mg/mL), obtained by dissolving the MTT powder in medium, was added to the cell cultures and incubated for 2 h at 37 °C; the formed crystals were melted with dimethylsulfoxide (DMSO) and used (200 µL of solution) to read the absorbance at 569 nm using a microplate reader (LabSystems-Multiskan Ascent 354 Microplate Reader, San Diego, CA, USA). Data are the mean of five independent experiments (an average of four readings was considered for each sample) and are expressed as the percent variation with respect to the absorbance at 569 nm recorded in untreated (control) cells. Standard deviations are represented by vertical bars. * Significantly different from untreated cells, *p* < 0.001.

Starting from the results of the time course experiments, we selected 48 h as the incubation time, sufficient to generate significant amounts of hA17–29 aggregates in solution. Data of Figure 2 show the dose response curve of the effects on cell survival of the addition to the culture medium of different concentrations of hA17–29. Results indicate that the cell incubation for 48 h with media supplemented with 1 or 3 µM hA17–29 did not significantly affect RBE4 viability, while higher concentrations (5 and 10 µM) determined a 18% and 25% decrease, respectively, of cell survival (*p* < 0.001 compared to untreated cells).

Since in the case of the MTT assay it is not possible to determine whether decreasing values are due to decreased cellular metabolic rate or increased cell death, to confirm that the decreased cell viability measured in our experiments was due to cell death, we performed additional experiments measuring the release of lactate dehydrogenase (LDH) in the culture media. The data in Table S1, showing the percent of increase in LDH equal in absolute values to the percent of decrease in absorbance at 569 nm (cell viability), clearly indicate that increasing concentrations of hA17–29 (1, 3, 5, and 10 µM) lead to an increase in cells death.

**Figure 3:**
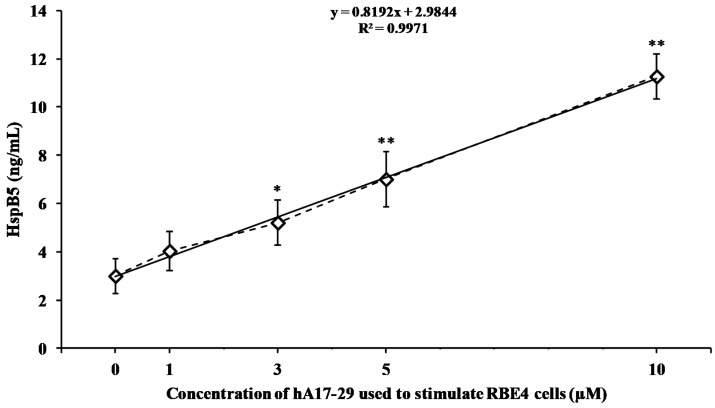
Effect of hA17–29 treatment on HspB5 secretion by RBE4 endothelial cells. RBE4 cells were stimulated for 48 h with different concentrations (1, 3, 5, and 10 µM) of freshly prepared hA17–29 peptide fragment. After 48 h, the medium was harvested and the amount of secreted HspB5 was quantified by ELISA according to the manufacturer instructions. The absorbance of each sample was measured with a spectrophotometer at 569 nm using a microplate reader (LabSystems-Multiskan Ascent 354 Microplate Reader, San Diego, CA, USA). The concentration of HspB5 in each sample was calculated through the use of a standard curve (from 1 ng/mL to 10 ng/mL). Data are the mean of three independent experiments (an average of four readings was considered for each sample). The dotted line is the trend of the experimental points (rhombus); the solid line is the best fitting straight line. * Significantly different from untreated (0 µM hA17–29), *p* < 0.01; ** significantly different from untreated, *p* < 0.001.

In order to evaluate the dose-dependent release of potentially protective factors as a physiological response of endothelial cells in response to stress, we measured the concentration of HspB5 in the medium either of untreated RBE4 or of RBE4 challenged for 48 h with increasing concentrations of hA17–29 (1, 3, 5, and 10 µM). As shown in Figure 3, a detectable amount of HspB5 was found even in the medium of RBE4 under basal conditions (untreated cells).

The minimal effective concentration of hA17–29 capable to induce a significant increase in the release of HspB5 was 3 µM. Using this concentration of hA17–29, we found that HspB5 released by RBE4 cells into the medium was 5.2 ng/mL, significantly higher (+79%, *p* < 0.01) than the concentration found in untreated RBE4 cells (2.9 ng/mL). The release of HspB5 during 48 h increased linearly by increasing the amount of hA17–29 added to the medium. Similar responses to stressing conditions were obtained when using the reference peptide Aβ25–35 (Figure S1).

This set of data, coupled to the results of hA17–29 cell viability and cell death in RBE4 cells (Figure 2 and Table S1), demonstrated that the concentration of 3 µM of hA17–29 is both the maximal dose with no significant cell toxicity within 48 h of incubation and the minimal dose causing significant release of potentially protective proteins (HspB5), during the same period of time. Therefore, the dose of 3 µM hA17–29 had the proper characteristics to obtain a CM after the challenge with RBE4. Protective effects of this CM were tested on the viability of SH-SY5Y cells challenged with a toxic concentration of hA17–29.

### 2.3. CM Derived from RBE4 Cells Counteracts SH-SY5Y Amyloid-Induced Toxicity

**Figure 4:**
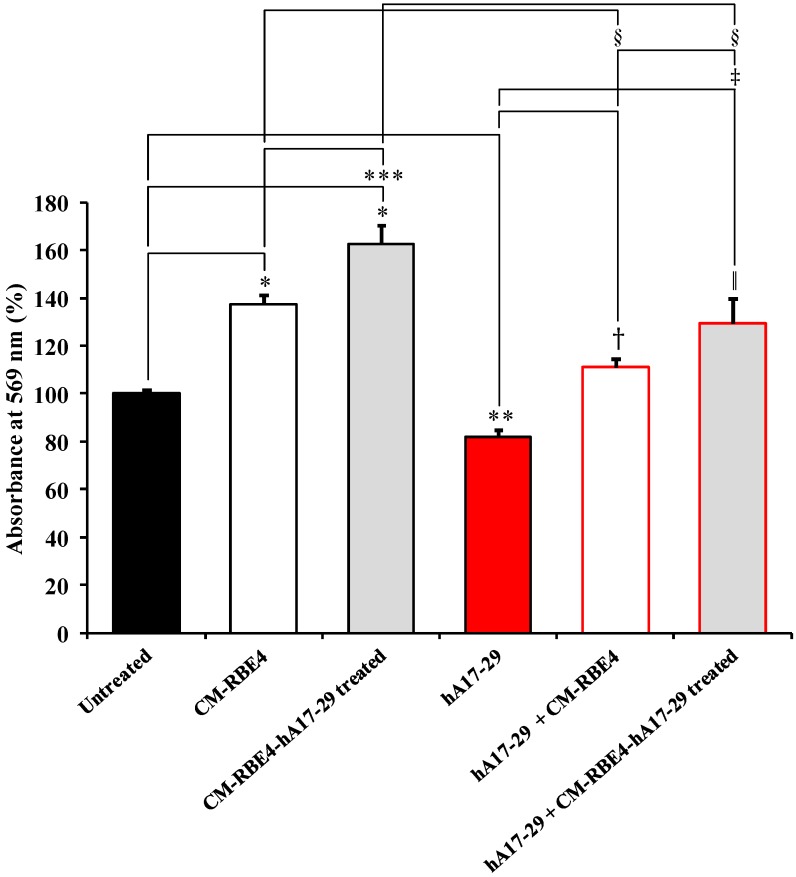
Conditioned medium (CM) derived from RBE4 cells counteracts SH-SY5Y hA17–29-induced toxicity. The MTT assay was carried out by measuring absorbance at 569 nm as described in Figure 2. Columns 2 and 3 show the effects on SH-SY5Y of the incubation for 48 h with CM obtained from untreated RBE4 (CM-RBE4) or RBE4 challenged with a sub-toxic (3 µM) concentration of hA17–29 (CM-RBE4-hA17–29). Column 4 clearly demonstrates the toxic effects due to the treatment with a high hA17–29 concentration (20 µM). Columns 5 and 6 depict the effects of both types of CM in counteracting, in SH-SY5Y, the decrease in absorbance induced by a toxic concentration (20 µM) of hA17–29. CM was diluted into fresh media, with a final concentration of 50%. Data are the mean of five independent experiments (an average of four readings was considered for each sample) and are expressed as the percent variation with respect to the absorbance at 569 nm detected in untreated (control) cultures. Standard deviations are represented by vertical bars. * Significantly different from untreated cells, *p* < 0.001; ** significantly different from untreated cells, *p* < 0.05; *** significantly different from CM-RBE4, *p* < 0.01; ^†^ significantly different from hA17–29, *p* < 0.01; ^‖^ significantly different from hA17–29, *p* < 0.001; ^‡^ significantly different from hA17–29 + CM-RBE4, *p* < 0.05; ^§^ significantly different from corresponding treatment with no hA17–29 (20 µM), *p* < 0.001.

Figure 4 depicts the effects on SH-SY5Y cells of the incubation for 48 h with CM from untreated RBE4 (CM-RBE4) or from RBE4 challenged with a sub-toxic concentration of hA17–29 (CM-RBE4-hA17–29) in presence or absence of a toxic treatment with hA17–29 (20 µM).

Both CMs produced a significant increase in absorbance at 569 nm on the MTT assay (*p* < 0.001 compared to control cells), with CM-RBE4-hA17–29 having the maximal effect (~+60%) and being significantly higher than that induced by CM-RBE4 (*p* < 0.01). This result suggests a metabolic rate and/or proliferation rate higher than control, induced by factors released in the medium by endothelial cells. Therefore, we assessed whether both CMs were capable to protect SH-SY5Y cells from the toxic effects produced by hA17–29. As shown in Figure 4, we found that SH-SY5Y cells challenged with 20 µM hA17–29 had decreased absorbance at 569 nm on the MTT assay by 21% (*p* < 0.01 compared to controls). Under these conditions, CM-RBE4 completely prevented hA17–29 toxicity (*p* < 0.01 compared to cells treated with hA17–29 only), with a slight increase in absorbance at 569 nm when compared to untreated (control) cells (~+10%). Even more evident effects were recorded when toxic hA17–29 was added to cell medium containing CM-RBE4-hA17–29. Absorbance at 569 nm on the MTT assay increased up to 130% (*p* < 0.01 compared to controls, *p* < 0.001 compared to cells treated with hA17–29 only, and *p* < 0.05 compared to cells treated with hA17–29 in presence of CM-RBE4 alone), indicating a combination of decreased cell death and increased metabolic and proliferative rates. It is worth underlining that a significant difference was observed when comparing CM-RBE4 and CM-RBE4-hA17–29 treatments (*p* < 0.001).

Since the effects of CM-RBE4-hA17–29 on hA17–29-treated SH-SY5Y cells were remarkably evident, we hypothesized that factors other than HspB5 were involved in the protective effects of CMs. To test the hypothesis that VEGF plays an essential role in the observed effects of CMs, we measured the absorbance at 569 on the MTT assay of SH-SY5Y cells incubated with the anti-Flk1 (blocking the VEGF receptor 2 (VEGFR2) and then receiving CM-RBE4 or CM-RBE4-hA17–29, both in the presence and in the absence of a toxic concentration of hA17–29 (20 µM). The results are summarized in Table 1. 

The pre-treatment for 2 h with anti-Flk1 only of untreated SH-SY5Y had no effects on absorbance at 569 nm (97.49% ± 4.89), over 48 h of incubation. When SH-SY5Y were pre-treated with anti-Flk1 and then challenged with CM-RBE4 or CM-RBE4-hA17–29, absorbance at 569 nm increased with respect to both untreated and anti-Flk1-treated cells (*p* < 0.01); however, it was significantly lower (−17.67%, *p* < 0.05 for CM-RBE4 and −29.41%, *p* < 0.001 for CM-RBE4-hA17–29) than that recorded in the absence of anti-Flk1 (Table 1). If compared with the corresponding treatment with no anti-Flk1, a slight decrease (−14.88%) was observed for SH-SY5Y cells pre-treated with anti-Flk1 and then challenged with CM-RBE4 and a toxic concentration of hA17–29 (20 µM). A greater difference (−25.35%, *p* < 0.01) was observed when comparing SH-SY5Y cells pre-treated with anti-Flk1 (and then challenged with CM-RBE4-hA17–29 and hA17–29) and the corresponding treatment with no anti-Flk1. The higher effect observed in cells pre-treated with anti-Flk1 and then with CM-RBE4-hA17–29 suggests the presence of a higher content of VEGF in the medium collected from RBE4 cells challenged with a sub-toxic concentration of hA17–29.

As shown in Table 2, the quantification of VEGF in the medium allowed to demonstrate that a sub-toxic concentration of hA17–29 (and Aβ25–35) caused a release of VEGF from RBE4, ultimately leading to a significant increase of this proteic factor in the cell medium.

It is worth underlining that the MTT experiments, as well as the experiment performed to quantify RBE4 VEGF levels in both CMs, when repeated with the active fragment Aβ25–35 as a reference amyloidogenic cytotoxic peptide, gave nearly identical results (Figure S2 and Table S2).

Since the MTT assay does not allow to clearly distinguish the decrease in cell death from increase in cell metabolic and proliferative rates, we measured the release of LDH in the culture media of all the different treatments. The results summarized in Table 3 show that the release of LDH of SH-SY5Y cells treated with both CMs (in presence or absence of anti-Flk1) was similar to untreated cells. Conversely, cells receiving hA17–29 treatment underwent a significant increase of LDH release compared to untreated cells (*p* < 0.001). Interestingly, the release of LDH from cells treated with hA17–29 + CM-RBE4 or hA17–29 + CM-RBE4-hA17–29 was significantly lower than that occurring from hA17–29-treated cells (*p* < 0.01); however, it was slightly higher than what was observed in the recorded untreated cells.

Similar results were obtained when measuring LDH release in experiments using Aβ25-35 as the reference amyloidogenic peptide (Table S3).

## 3. Discussion

In the last decades, different studies have demonstrated the toxic effects of amylin on neuron-like and pancreatic β-cells [30,31,32]. Few of them investigated the possibility that low amylin concentrations might have beneficial effects especially at the brain level [33,34]. In this study, we explored the hypothesis that sub-toxic concentrations of the amylin fragment hA17–29 activate, when present into its amyloidogenic aggregate form, a beneficial “cross-talk” between endothelial cells of the brain vasculature and neurons. We used RBE4–SH-SY5Y cells [28,29] to reproduce an experimental model mimicking a functional neurovascular unit [35], and interestingly, we found that in our experiments the benefit was represented by the release from endothelial cells of neuroprotective factors able to counteract amyloid-induced cell death of neuron-like cell cultures. 

When monitoring hA17–29 amyloid fibril formation using the Th-T assay, we observed that the concentration of amyloid fibrils was significantly different from zero time only after 12, 24, and 48 h (Figure 1). The longest incubation time was then used to challenge RBE4 cells with increasing hA17–29 concentrations, finding that 3 µM was the maximal non-toxic concentration causing no change in absorbance at 569 nm on the MTT test (Figure 2). Interestingly, we found that this non-toxic hA17–29 concentration was able to stimulate the secretion of biologically-relevant concentrations of HspB5 in the medium (Figure 3). Therefore, by combining the information obtained from these three sets of experiments, we selected the optimal incubation condition (48 h) to generate RBE4 CM, then used it to treat SH-SY5Y cells and verify the hypothesized intercellular “cross-talk” between endothelial cells and neuron-like cells. 

The decrease in absorbance at 569 nm on the MTT test, observed when challenging SH-SY5Y cells with toxic hA17–29 (20 µM) (Figure 4), was counteracted by the co-treatment with CMs, with SH-SY5Y cells showing a +48% increase when co-treated with CM-RBE4-hA17–29 (*p* < 0.001 compared to controls). By combining results of the MTT (Figure 4) and LDH (Table 3) assays it was possible to highlight that the observed increase in absorbance at 569 nm on the MTT test, measured for cells treated with hA17–29 + CMs, was due to a decrease in cell death (cell protection; lower amount of LDH released) and, most likely, by an increase in cell metabolism and proliferation. In fact, cells treated with hA17–29 + CM-RBE4 or hA17–29 + CM-RBE4-hA17–29 had significantly lower cell death compared to hA17–29-treated cells, and higher cell death when compared to untreated cells.

Several studies pointed out that HspB5, primarily found as a major structural protein for the maintenance of ocular lens transparency [36,37], can be induced as a principal member of the mammalian HspBs in non-lenticular tissues [38,39]. As showed by the results obtained by Brady et al. [40], who employed mice null for HspB5, this small protein is implicated in several physiological activities, and its decreased or missing expression correlates with aging deficits at the peripheral nervous system level. HspB5 can be overexpressed in different neurodegenerative disorders including AD [41,42,43,44]. The enhanced expression and secretion of HspB5 have been observed in different cell types, including endothelial cells [45]. Therefore, results obtained in the present study using CM to reduce amylin toxicity might be explained by the presence of HspB5 in the CM (Figure 3), and our data are in agreement with literature data showing how an increased production of HspBs, and specifically HspB5, prevents cell death [46,47,48]. In particular, part of the protective effects observed in SH-SY5Y cells may depend on the ability of HspB5 in counteracting the aggregation of amyloid peptides [49,50], and thus, the formation of more toxic species such as the oligomers [26]. It is important to point out that the increase in HspB5 production by endothelial cells as a consequence of increasing concentrations of stressor (Figure 2) was able to counteract amyloid-induced cell death only at low hA17–29 concentrations (≤3 µM), since, at higher hA17–29 concentrations (≥5 µM), the HspB5 protection was overcome by hA17–29 toxicity.

However, it has been well established that endothelial cells produce, both in vitro and in vivo, numerous trophic factors involved in brain homeostasis [51,52]. Among them, VEGF is one of the most abundant endothelial secretory factors that have been found to be regulated by HspB5. In fact, when a decrease in the phosphorylation of HspB5 is observed, a concomitant decrease in VEGF expression occurs (because of malfunctioning of the endoplasmic reticulum) and imperfect angiogenesis and increased endothelial apoptosis have been detected [22].

The connection between these two endothelial secretory proteic factors prompted us to test the possibility that the beneficial effects of CM against cytotoxicity caused by 20 µM hA17–29 in SH-SY5Y cells might be due to the synergistic action of HspB5 and VEGF, simultaneously secreted in the medium by RBE4 and challenged with a sub-toxic hA17–29 concentration (3 µM). Since data from literature showed that SH-SY5Y cells express VEGF receptor [26,53,54], we verified our hypothesis by pre-treating SH-SY5Y with the antibody anti-VEGFR2 receptor (anti-Flk1), prior to the addition of CM-RBE4 or CM-RBE4-hA17–29, and the subsequent treatment with a toxic concentration of hA17–29 (20 µM). The results clearly showed that the pre-treatment with anti-Flk1 (Table 1) produced a significant decrease in the protective effects caused by both CM-RBE4 and CM-RBE4-hA17–29, thus, strongly suggesting that the release of VEGF significantly contributes as the additional protective factor to the overall increased resistance of neuron-like SH-SY5Y cells towards amylin cytotoxic concentrations. The aforementioned data were strongly corroborated by the direct quantification of VEGF in CM-RBE4 and CM-RBE4-hA17–29 (Table 2). This result strengthens the hypothesis that stressed RBE4 cells secrete more VEGF if compared to untreated (unstressed) RBE4 cells and are in agreement with previous studies showing that endothelial RBE4 cells increase VEGF expression and secretion under stress [55] even in presence of low concentration of amyloid proteins [56].

Alongside the evidences showing the protective effect of both CM-RBE4 or CM-RBE4-hA17–29 (or CM-RBE4-Aβ25–35) against amyloid toxicity, it is important to point out the effects exerted by both CMs on SH-SY5Y cells under normal condition (columns 2 and 3 of Figure 4 and Figure S2). Even in the absence of a sub-toxic stimulus (hA17–29 or Aβ25–35), the medium conditioned by the endothelial cells (CM-RBE4) was able to significantly increase (compared to controls) the absorbance at 569 nm measured on the MTT test, suggesting an increase of metabolic rate and/or proliferation rate compared to cells under normal condition (control untreated cells). In addition to the previously mentioned HspB5 chaperone, one of the factors definitely involved in the observed protective effects is the VEGF, as supported by its quite high level (301.75 ± 47.72 pg/mL) (Table 2) secreted in the medium by endothelial cells under normal conditions. This “physiological” secretion of VEGF by RBE4 cells and its effect on SH-SY5Y cells are in accordance with other recent studies published in this field [55,57]. As highlighted by Rosenstein et al. [57], within the central nervous system (CNS) VEGF is able to promote neurogenesis, has trophic effects on neurons and glia, supports neuronal migration in the developing CNS, and is essential for neuroprotection in adults. Restin et al. [55] demonstrated that in pathological conditions, such as severe hypoxia, the basal production of VEGF can reach levels more than ten times higher (e.g., 2597 ± 888) compared to untreated cells. In accordance to these results, our study strongly suggests a control mechanism switching VEGF endocytosis from endogenous basal activities (e.g., neurotrophic effects; columns 2 and 3 of Figure 4 and Figure S2) to neuroprotection (columns 5 and 6 of Figure 4 and Figure S2).

Taking into account the well-known dependent relationship between HspB5 and VEGF, the increased release of this HspB by endothelial cells, under our experimental conditions, may be fundamental for VEGF proper folding [22,24]. This effect might possibly enhance VEGF bioavailability, ultimately leading to greater protective and proliferative effects on SH-SY5Y cells (Table 1 and Table 3). Herrán et al. [21] have shown that VEGF prevents amyloid-induced neurotoxicity enhancing neuronal proliferation. Additionally, the results of the present study allow to hypothesize that VEGF increases cell metabolic activity and proliferation by preventing cell cycle deregulation, a primary event in amyloid-related pathologies such as AD [58,59,60].

Both HspB5 and VEGF are low molecular weight proteins (of approximately 19 and 22 kDa, respectively). While VEGF is a secretory protein with different types of cells as targets and is increasingly released by endothelial cells under different stressing conditions [61,62], HspB5 intracellularly exploits its chaperone activity, and is not considered a secretory protein [63], even though some research have showed a release of HspB5 under various experimental conditions [64,65]. Results reported in the present study indicate that human amylin, in concentration not causing change in endothelial cell viability, represents a sufficient stressor to stimulate RBE4 cells in releasing both HspB5 and VEGF in the extracellular environment. Remarkably, nearly identical results have been obtained when using the reference β-amyloid peptide fragment Aβ25–35 in place of hA17–29. It is known that Aβ peptides exert their neurotoxic effects binding to amylin receptor [66]. We cannot exclude that Aβ25–35, the active fragment of Aβ1-42, elicits neuroprotective effects in SH-SY5Y cells through the activation of amylin receptors in endothelial cells and the following release of neuroprotective factors such as HspB5 and VEGF.

## 4. Materials and Methods

### 4.1. Chemicals

All chemicals used in this work were of analytical grade and purchased from Sigma (St. Louis, MO, USA), unless specified otherwise. SH-SY5Y cells (CRL-2266™) were purchased from ATCC (Manassas, VA, USA), while the RBE4 cells were kindly provided by Dr. Lopalco’s laboratory, University of Kansas (Lawrence, KS, USA). Peptide fragments hA17–29 and Aβ25–35 were purchased from Life Technologies (Monza, Italy) and Bachem Distribution Services GmbH (Weil am Rhein, Germany), respectively. Fetal bovine serum (FBS), plasma-derived serum (PDS), penicillin/streptomycin solution, Ham’s F10, F12, and Dulbecco’s Modified Eagle’s Medium (DMEM) were purchased from Lonza (Walkersville, MD, USA). Antibody Flk-1 (C-1158): sc-504 was obtained from Santa Cruz Biotechnology (Heidelberg, Germany). The HspB5 Immunoset was purchased from Enzo Life Sciences (Farmingdale, NY, USA). The VEGF Quantikine ELISA Kit was obtained from R&D Systems (Minneapolis, MN, USA).

### 4.2. Peptide Fragments Monomerization and Aggregation Studies

The peptide fragments (hA17–29 and the reference peptide Aβ25–35) monomerization was previously described in detail in [26]. Peptide fragments hA17–29 and Aβ25–35 were monomerized after incubation overnight in sealed vials with a 1 mM final concentration of 1,1,1,3,3,3-hexafluoro-2-propanol (HFIP). The next day, HFIP was evaporated under a gentle stream of nitrogen and the dried peptide fragments were used either immediately or stored at −80 °C. Immediately before starting the aggregation studies, monomeric peptides were dissolved in DMSO, and subsequently diluted using PBS at pH 7.4, up to a concentration (100 µM) suitable to carry out structurally defined and reproducible aggregation experiments [26,67,68]. The incubation of hA17–29 and Aβ25–35 at 37 °C was protracted for 0, 1, 3, 6, 12, 24, and 48 h.

The aggregation process of the hA17–29 and Aβ25–35 peptide fragments was followed by the well-known Th-T assay, as previously described [26]. The parameters used to detect the fluoresce coming from the formation of aggregated structures were selected according to LeVine 3rd [69]. A LabSystems-Multiskan Ascent 354 Microplate Reader (San Diego, CA, USA) was employed to read the fluorescence in a 96-well plate. The final fluorescence values were calculated by subtracting the fluorescence produced by control solutions (Th-T alone and aggregates in solution in absence of Th-T).

### 4.3. Rat Brain Endothelial (RBE4) and Human Neuroblastoma (SH-SY5Y) Cell Cultures 

RBE4 cells were cultured in Ham’s F10 medium supplemented with 20% (*v*/*v*) PDS, 2 mM L-glutamine, penicillin (50 U/mL), streptomycin (50 µg/mL), 0.5% of endothelial cell growth supplement (ECGS), and 2% of heparin. The cells were maintained in a humidified environment at 37 °C and 5% CO_2_ and cultured in 25 cm^2^ culture flasks pre-coated with collagen. The medium was changed twice a week and cells were split at about 90–95% confluence.

SH-SY5Y cells were cultured in a mixture of the same amount of DMEM and F12 media enriched with 10% (*v*/*v*) FBS. The final concentrations of penicillin and streptomycin in the medium were 50 U/mL and 50 µg/mL, respectively. The cells were maintained in a humidified environment at 37 °C and 5% CO_2_ and cultured in 75 cm^2^ culture flasks. The medium was replaced twice a week and cells were split at about 75–80% confluence.

### 4.4. RBE4 Cells Stimulation and Quantification of HspB5 and VEGF in Conditioned Medium

Before collecting RBE4CM, cells previously cultured in 25 cm^2^ culture flasks pre-coated with collagen were washed using 5 mL of cold 10 mM PBS at pH 7.4, harvested using 2.5 mL of trypsin-EDTA solution (0.25% Trypsin/0.53 mM EDTA in Hanks Balanced Salt Solution without calcium or magnesium), and seeded in 6-well plates pre-coated with collagen at a density of 3 × 10^5^ cells/well. In order to evaluate the ability of hA17–29 peptide fragment in modulating the release of HspB5 into culture medium, RBE4 cells were stimulated for 48 h with different concentrations (1, 3, 5, and 10 µM) of hA17–29 as soon as the appropriate confluence was reached. At the end of the stimulation process the medium was collected for LDH, HspB5, and VEGF dosage while cell viability was assessed employing the MTT assay [26,70].

Release of HspB5 into culture medium was measured using the immunoenzymatic ELISA kit according to the manufacturer instructions. Briefly, 100 µL of standards with known concentration and an equal amount of medium samples (previously centrifuged for 10 min at 1000× *g* to remove any particulate material) were incubated in microplate wells pre-coated with an antibody specific for HspB5. After incubation, biotinylation, and a conjugation with streptavidin-horseradish peroxidise plates were incubated for 30 min at 37 °C with 3,3′,5,5′-tetramethylbenzidine. The reaction was stopped by the addition of 100 µL of HCl 1 N solution and the absorbance of the resulting yellow product was measured with a spectrophotometer at 569 nm using a microplate reader (LabSystems-Multiskan Ascent 354 Microplate Reader, San Diego, CA, USA). Using this protocol, the standard curve for HspB5 was linear and ranged from 1 ng/mL to 10 ng/mL. The release of HspB5 by RBE4 cells following hA17–29 treatment was compared with the quantity of HspB5 released by the same cell type challenged with equal concentrations of the reference peptide Aβ25–35.

Quantification of VEGF content in CM from untreated or REB4 cells treated with a sub-toxic concentration of hA17–29 (or Aβ25–35) was performed using the VEGF Quantikine ELISA according to manufacturer’s instructions. Briefly, the addition of 50 µL of assay diluent to each well was followed by the addition of an equal amount of standard or CM samples. The plate was then covered with a plate sealer and incubated at room temperature for 2 h on a microplate shaker. After five washing steps, 100 µL of conjugate was added to each well and the plate was covered with a new plate sealer and incubated at room temperature for 1 h under shaking. Subsequently, substrate solution addition (100 µL), incubated at room temperature (30 min) in the dark, and addition of stop solution (100 µL) were performed to each well. The absorbance was measured within 30 min by a microplate reader (LabSystems-Multiskan Ascent 354 Microplate Reader, San Diego, CA, USA) at a wavelength of 450 nm. Wavelength correction was performed by subtracting measurements at 570 nm from the 450 nm readings.

### 4.5. RBE4 Conditioned Media Counteract SH-SY5Y Amyloid-Induced Toxicity: the Role of VEGF

On the day prior to treatment, SH-SY5Y cells were harvested and seeded in 48-well plates at a density of 25 × 10^3^ cells/well. As soon as the appropriate confluence was reached, the culture medium of untreated cells was replaced with fresh medium while for all other treatments the culture medium was replaced with fresh medium containing the appropriate treatments.

To evaluate the effects of the CM obtained from untreated and RBE4 cells stimulated with a sub-toxic concentration (3 µM) of hA17–29 on SH-SY5Y, neuroblastoma cells were treated for 48 h with a toxic concentration (20 µM) of hA17–29 [26] in presence or absence of CM obtained from untreated (CM-RBE4) or hA17–29-treated (CM-RBE4-hA17–29 treated) RBE4 cells. In each well, when present, the final concentration of CM-RBE4 or CM-RBE4-hA17–29 treated was equal to 50% of the total medium volume (50% SH-SY5Y medium + 50% CM-RBE4 or CM-RBE4-hA17–29 treated). For SH-SY5Y treatment, we chose to use the medium obtained by stimulating RBE4 cells with 3 µM hA17–29, since the challenge with higher concentrations of amylin fragment (≥5 µM) is known to be significantly toxic to endothelial cells. To ascertain the contribution that VEGF may play in preventing amyloid-induced toxicity (20 µM), cells were pre-treated for 2 h with the antibody anti-Flk1 (VEGFR2). Anti-Flk1, at the final concentration of 2 µg/mL, was added to each appropriate well in order to saturate the receptors for VEGF. Following 2 h pre-treatment, the medium was replaced with fresh medium (Control anti-Flk1) or with medium containing the peptide fragments and/or the CM, after which cells were incubated for an additional 48 h. At the end of incubation, the MTT assay and the LDH assay were carried out to analyze SH-SY5Y cells and SH-SY5Y medium, respectively, as described for RBE4 cells.

The well-known peptide fragment Aβ25–35 was employed as a reference peptide to compare the MTT and LDH data obtained using the hA17–29 peptide fragment.

### 4.6. Statistical Analysis

The statistical analysis employed to study hA17–29 (and the reference peptide Aβ25–35) activity was the same already employed and described elsewhere [71,72]. Normal data distribution was tested using the Kolmogorov-Smirnov test. The within-group comparison was performed by a one-way analysis of variance (ANOVA). Differences across groups were estimated by a two-way ANOVA. Fisher’s protected least square was used as a post hoc test. Only two-tailed p-values of less than 0.05 were considered statistically significant [73].

## 5. Conclusions

This study demonstrated that amylin (and β-amyloid) might play an important physiological role in promoting the release of neuroprotective factors, namely HspB5 and VEGF, from endothelial cells of the capillary neurovasculature. The data presented in this study confirm the existence of a “cross-talk” between endothelial and neuron-like cells and the relevance of a physiological role of amylin and β-amyloid, such as the ability to promote the release of neuroprotective factors from endothelial cells. Further studies are needed to better understand the role of this cross-talk in the onset and development of amyloid-related neurodegenerative disorders and its relevance for drug discovery processes in AD.

Recent studies have demonstrated that amylin levels are reduced in AD patients [33] and pramlintide, an amylin analogue recently approved for the treatment of diabetes, is known to exert neuroprotective effects in experimental models of AD [74,75]. When considering this evidence, we believe that the data presented in this manuscript might be relevant to better understand the neuroprotective potential of human amylin, as well as the therapeutic potential of amylin analogues in experimental models of AD.

## Figures and Tables

**Table 1:** Effect of anti-Flk1 antibody pre-treatment (2 µg/mL—2 h) on SH-SY5Y cells.

Cell Treatment	Absorbance at 569 nm (%) in Absence of Anti-Flk1	Absorbance at 569 nm (%) in Presence of Anti-Flk1	Difference
CM-RBE4	134.27 (5.98)	116.55 ^a^ (5.23)	−17.72
CM-RBE4-hA17–29	158.88 (8.18)	129.47 ^b^ (6.99)	−29.41
hA17–29 + CM-RBE4	113.79 (4.75)	98.90 ^d^ (4.18)	−14.88
hA17–29 + CM-RBE4-hA17–29	126.38 (7.22)	101.03 ^c,e^ (3.75)	−25.35

The MTT assay was carried out by measuring absorbance at 569 nm as described in Figure 2. CM was diluted into fresh media to a final concentration of 50%. The concentration of hA17–29 was 20 µM. Data are the mean of three independent experiments (an average of four readings was considered for each sample) and are expressed as the percent variation with respect to the absorbance detected in untreated (control) cultures. Standard deviations are reported in parentheses. Difference = Absorbance at 569 nm (%) in absence of anti-Flk1—Absorbance at 569 nm (%) in presence of anti-Flk1. ^a^ Significantly different from corresponding treatment with no anti-Flk1, *p* < 0.05; ^b^ significantly different from corresponding treatment with no anti-Flk1, *p* < 0.001; ^c^ significantly different from corresponding treatment with no anti-Flk1, *p* < 0.01; ^d^ significantly different from corresponding treatment with no hA17–29, *p* < 0.05; ^e^ significantly different from corresponding treatment with no hA17–29, *p* < 0.001.

**Table 2:** VEGF levels measured in conditioned media obtained from untreated or RBE4 cells treated with a sub-toxic concentration of hA17–29 (or Aβ25–35).

Cell Treatment	VEGF Levels (pg/mL)	% of Increase Compared to CM-RBE4
CM-RBE4	301.75 ± 47.72	//
CM-RBE4-hA17–29	862.25 ± 72.2 ^a,b^	+286
CM-RBE4-Aβ25–35	709.50 ± 82.20 ^a^	+235

Quantification of VEGF content in conditioned medium (CM) was performed using the VEGF Quantikine ELISA according to manufacturer’s instructions. The absorbance of each well was measured within 30 min by a microplate reader (LabSystems-Multiskan Ascent 354 Microplate Reader, San Diego, CA, USA) at a wavelength of 450 nm. Wavelength correction was performed by subtracting the measurements at 570 nm from the 450 nm readings. Values are the mean of four different experiments and are expressed as picogram (pg)/mL of medium analyzed. The concentration of hA17–29 and Aβ25–35 is 3 µM. Standard deviations are reported in parenthesis. ^a^ Significantly different from CM-RBE4, *p* < 0.001; ^b^ significantly different from CM-RBE4-Aβ25–35, *p* < 0.05.

**Table 3:** SH-SY5Y cells death induced by the treatment for 48 h with a high concentration of hA17–29 and effect of conditioned media.

Cell Treatment	LDH Release (% of Controls) in Absence of Anti-Flk1	LDH Release (% of Controls) in Presence of Anti-Flk1
hA17–29	128.98 ^a^ (3.82)	NA
CM-RBE4	97.90 (2.31)	102.75 (4.41)
CM-RBE4-hA17–29	99.97 (1.97)	100.84 (2.73)
hA17–29 + CM-RBE4	111.92 ^b^ (2.54)	116.09 (3.35)
hA17–29 + CM-RBE4-hA17–29	106.52 ^b^ (3.84)	113.84 (5.18)

CM was diluted into fresh media to a final concentration of 50%. The concentration of hA17–29 was 20 µM. Cell death was determined using the LDH assay as described in Table S1. Data are the mean of four independent experiments (an average of four readings was considered for each sample) and are expressed as the percent variation with respect to the LDH release recorded in untreated (control) cultures. Standard deviations are reported in parentheses. NA = Not assessed. ^a^ Significantly different from untreated cells, *p* < 0.001; ^b^ significantly different from hA17–29, *p* < 0.01.

## Supplementary Materials

Supplementary materials can be found at http://www.mdpi.com/1422-0067/19/11/3659/s1. Figure S1. Effect of Aβ25–35 treatment on heat shock protein B5 (HspB5) secretion by RBE4 endothelial cells. Figure S2. Conditioned medium (CM) derived from RBE4 cells counteracts SH-SY5Y Aβ25–35-induced toxicity. Table S1. RBE4 cells death induced by increasing concentrations of hA17-29. Table S2. Effect of anti-Flk1 antibody pre-treatment (2 µg/mL - 2 h) on SH-SY5Y cells. Table S3. SH-SY5Y cells death induced by the treatment for 48 h with a high concentration of Aβ25–35 and effect of conditioned media.

## References

[1] Ahren B., Oosterwijk C., Lips C.J., Hoppener J.W. (1998). Transgenic overexpression of human islet amyloid polypeptide inhibits insulin secretion and glucose elimination after gastric glucose gavage in mice. Diabetologia.

[2] Martin C. (2006). The physiology of amylin and insulin. Diabetes Educ..

[3] Sanke T., Bell G.I., Sample C., Rubenstein A.H., Steiner D.F. (1988). An islet amyloid peptide is derived from an 89-amino acid precursor by proteolytic processing. J. Biol. Chem..

[4] Marzban L., Trigo-Gonzalez G., Verchere C.B. (2005). Processing of pro-islet amyloid polypeptide in the constitutive and regulated secretory pathways of beta cells. Mol. Endocrinol..

[5] Kodali R., Wetzel R. (2007). Polymorphism in the intermediates and products of amyloid assembly. Curr. Opin. Struct. Biol..

[6] Jaikaran E.T., Clark A. (2001). Islet amyloid and type 2 diabetes: From molecular misfolding to islet pathophysiology. Biochim. Biophys. Acta.

[7] Lorenzo A., Razzaboni B., Weir G.C., Yankner B.A. (1994). Pancreatic islet cell toxicity of amylin associated with type-2 diabetes mellitus. Nature.

[8] Jackson K., Barisone G.A., Diaz E., Jin L.W., DeCarli C., Despa F. (2013). Amylin deposition in the brain: A second amyloid in Alzheimer disease?. Ann. Neurol..

[9] Wijesekara N., Ahrens R., Sabale M., Wu L., Ha K., Verdile G., Fraser P.E. (2017). Amyloid-beta and islet amyloid pathologies link Alzheimer’s disease and type 2 diabetes in a transgenic model. FASEB J..

[10] Caraci F., Tascedda F., Merlo S., Benatti C., Spampinato S.F., Munafo A., Leggio G.M., Nicoletti F., Brunello N., Drago F. (2016). Fluoxetine prevents abeta1-42-induced toxicity via a paracrine signaling mediated by transforming-growth-factor-beta1. Front. Pharmacol..

[11] Polazzi E., Contestabile A. (2003). Neuron-conditioned media differentially affect the survival of activated or unstimulated microglia: Evidence for neuronal control on apoptotic elimination of activated microglia. J. Neuropathol. Exp. Neurol..

[12] Arai K., Lo E.H. (2009). An oligovascular niche: Cerebral endothelial cells promote the survival and proliferation of oligodendrocyte precursor cells. J. Neurosci..

[13] Ghosh J.G., Shenoy A.K.Jr., Clark J.I. (2007). Interactions between important regulatory proteins and human alphab crystallin. Biochemistry.

[14] Kamradt M.C., Chen F., Cryns V.L. (2001). The small heat shock protein alpha b-crystallin negatively regulates cytochrome c- and caspase-8-dependent activation of caspase-3 by inhibiting its autoproteolytic maturation. J. Biol. Chem..

[15] Kamradt M.C., Lu M., Werner M.E., Kwan T., Chen F., Strohecker A., Oshita S., Wilkinson J.C., Yu C., Oliver P.G. (2005). The small heat shock protein alpha b-crystallin is a novel inhibitor of trail-induced apoptosis that suppresses the activation of caspase-3. J. Biol. Chem..

[16] Morozov V., Wawrousek E.F. (2006). Caspase-dependent secondary lens fiber cell disintegration in alphaa-/alphab-crystallin double-knockout mice. Development.

[17] Ferrara N., Gerber H.P., LeCouter J. (2003). The biology of vegf and its receptors. Nat. Med..

[18] Fischer S., Clauss M., Wiesnet M., Renz D., Schaper W., Karliczek G.F. (1999). Hypoxia induces permeability in brain microvessel endothelial cells via vegf and no. Am. J. Physiol..

[19] Fischer S., Wobben M., Marti H.H., Renz D., Schaper W. (2002). Hypoxia-induced hyperpermeability in brain microvessel endothelial cells involves vegf-mediated changes in the expression of zonula occludens-1. Microvasc. Res..

[20] Kovacs Z., Ikezaki K., Samoto K., Inamura T., Fukui M. (1996). Vegf and flt. Expression time kinetics in rat brain infarct. Stroke.

[21] Herrán E., Perez-Gonzalez R., Igartua M., Pedraz J.L., Carro E., Hernandez R.M. (2013). Vegf-releasing biodegradable nanospheres administered by craniotomy: A novel therapeutic approach in the app/ps1 mouse model of Alzheimer’s disease. J. Control. Release.

[22] Kase S., He S., Sonoda S., Kitamura M., Spee C., Wawrousek E., Ryan S.J., Kannan R., Hinton D.R. (2010). Alphab-crystallin regulation of angiogenesis by modulation of vegf. Blood.

[23] Van de Schootbrugge C., Bussink J., Span P.N., Sweep F.C., Grénman R., Stegeman H., Pruijn G.J., Kaanders J.H., Boelens W.C. (2013). αB-crystallin stimulates VEGF secretion and tumor cell migration and correlates with enhanced distant metastasis in head and neck squamous cell carcinoma. BMC Cancer.

[24] Kerr B.A., Byzova T.V. (2010). Alphab-crystallin: A novel vegf chaperone. Blood.

[25] Pappalardo G., Milardi D., Magri A., Attanasio F., Impellizzeri G., La Rosa C., Grasso D., Rizzarelli E. (2007). Environmental factors differently affect human and rat iapp: Conformational preferences and membrane interactions of iapp17-29 peptide derivatives. Chemistry.

[26] Caruso G., Distefano D.A., Parlascino P., Fresta C.G., Lazzarino G., Lunte S.M., Nicoletti V.G. (2017). Receptor-mediated toxicity of human amylin fragment aggregated by short- and long-term incubations with copper ions. Mol. Cell Biochem..

[27] Mazzaglia A., Micali N., Scolaro L.M., Attanasio F., Magri A., Pappalardo G., Villari V. (2010). Aggregation properties of the peptide fragments derived from the 17–29 region of the human and rat iapp: A comparative study with two peg-conjugated variants of the human sequence. J. Phys. Chem. B.

[28] Balbuena P., Li W., Magnin-Bissel G., Meldrum J.B., Ehrich M. (2010). Comparison of two blood-brain barrier in vitro systems: Cytotoxicity and transfer assessments of malathion/oxon and lead acetate. Toxicol. Sci..

[29] Toimela T., Maenpaa H., Mannerstrom M., Tahti H. (2004). Development of an in vitro blood-brain barrier model-cytotoxicity of mercury and aluminum. Toxicol. Appl. Pharmacol..

[30] Sinopoli A., Magri A., Milardi D., Pappalardo M., Pucci P., Flagiello A., Titman J.J., Nicoletti V.G., Caruso G., Pappalardo G. (2014). The role of copper(ii) in the aggregation of human amylin. Metallomics.

[31] Konarkowska B., Aitken J.F., Kistler J., Zhang S., Cooper G.J. (2006). The aggregation potential of human amylin determines its cytotoxicity towards islet beta-cells. FEBS J..

[32] Akter R., Cao P., Noor H., Ridgway Z., Tu L.H., Wang H., Wong A.G., Zhang X., Abedini A., Schmidt A.M. (2016). Islet amyloid polypeptide: Structure, function, and pathophysiology. J. Diabetes Res..

[33] Adler B.L., Yarchoan M., Hwang H.M., Louneva N., Blair J.A., Palm R., Smith M.A., Lee H.G., Arnold S.E., Casadesus G. (2014). Neuroprotective effects of the amylin analogue pramlintide on Alzheimer’s disease pathogenesis and cognition. Neurobiol. Aging.

[34] Qiu W.Q., Zhu H. (2014). Amylin and its analogs: A friend or foe for the treatment of Alzheimer’s disease?. Front. Aging Neurosci..

[35] Xue Q., Liu Y., Qi H., Ma Q., Xu L., Chen W., Chen G., Xu X. (2013). A novel brain neurovascular unit model with neurons, astrocytes and microvascular endothelial cells of rat. Int. J. Biol. Sci..

[36] Muchowski P.J., Bassuk J.A., Lubsen N.H., Clark J.I. (1997). Human alphab-crystallin. Small heat shock protein and molecular chaperone. J. Biol. Chem..

[37] Horwitz J., Bova M.P., Ding L.L., Haley D.A., Stewart P.L. (1999). Lens alpha-crystallin: Function and structure. Eye (Lond).

[38] Lowe J., McDermott H., Pike I., Spendlove I., Landon M., Mayer R.J. (1992). Alpha b crystallin expression in non-lenticular tissues and selective presence in ubiquitinated inclusion bodies in human disease. J. Pathol..

[39] Singh B.N., Rao K.S., Rao Ch M. (2010). Ubiquitin-proteasome-mediated degradation and synthesis of myod is modulated by alphab-crystallin, a small heat shock protein, during muscle differentiation. Biochim. Biophys. Acta.

[40] Brady J.P., Garland D.L., Green D.E., Tamm E.R., Giblin F.J., Wawrousek E.F. (2001). AlphaB-crystallin in lens development and muscle integrity: A gene knockout approach. Investig. Ophthalmol. Vis. Sci..

[41] Ito H., Kamei K., Iwamoto I., Inaguma Y., Kato K. (2001). Regulation of the levels of small heat-shock proteins during differentiation of c2c12 cells. Exp. Cell Res..

[42] Renkawek K., Voorter C., Bosman G., van Workum F., De Jong W. (1994). Expression of HspB5 in Alzheimer’s disease. Acta Neuropathol..

[43] Diokmetzidou A., Soumaka E., Kloukina I., Tsikitis M., Makridakis M., Varela A., Davos C.H., Georgopoulos S., Anesti V., Vlahou A. (2016). Desmin and alphab-crystallin interplay in the maintenance of mitochondrial homeostasis and cardiomyocyte survival. J. Cell Sci..

[44] Oliveira A.O., Osmand A., Outeiro T.F., Muchowski P.J., Finkbeiner S. (2016). Alphab-crystallin overexpression in astrocytes modulates the phenotype of the bachd mouse model of Huntington’s disease. Hum. Mol. Genet..

[45] Golenhofen N., Ness W., Wawrousek E.F., Drenckhahn D. (2002). Expression and induction of the stress protein alpha-b-crystallin in vascular endothelial cells. Histochem. Cell Biol..

[46] Tang S., Yin B., Song E., Chen H., Cheng Y., Zhang X., Bao E., Hartung J. (2016). Aspirin upregulates alphab-crystallin to protect the myocardium against heat stress in broiler chickens. Sci. Rep..

[47] Zhou P., Kannan R., Spee C., Sreekumar P.G., Dou G., Hinton D.R. (2014). Protection of retina by alphab crystallin in sodium iodate induced retinal degeneration. PLoS ONE.

[48] Mehlen P., Kretz-Remy C., Preville X., Arrigo A.P. (1996). Human hsp27, drosophila hsp27 and human alphab-crystallin expression-mediated increase in glutathione is essential for the protective activity of these proteins against tnfalpha-induced cell death. EMBO J..

[49] Raman B., Ban T., Sakai M., Pasta S.Y., Ramakrishna T., Naiki H., Goto Y., Rao C.M. (2005). AlphaB-crystallin, a small heat-shock protein, prevents the amyloid fibril growth of an amyloid beta-peptide and beta2-microglobulin. Biochem. J..

[50] Liu Z., Zhang S., Li D., Liu C. (2017). A Structural View of αB-crystallin Assembly and Amyloid Aggregation. Protein Pept. Lett..

[51] Wang H., Ward N., Boswell M., Katz D.M. (2006). Secretion of brain-derived neurotrophic factor from brain microvascular endothelial cells. Eur. J. Neurosci..

[52] Feng S., Zhuang M., Wu R. (2012). Secretion of nerve growth factor, brain-derived neurotrophic factor, and glial cell-line derived neurotrophic factor in co-culture of four cell types in cerebrospinal fluid-containing medium. Neural. Regen. Res..

[53] Meister B., Grunebach F., Bautz F., Brugger W., Fink F.M., Kanz L., Mohle R. (1999). Expression of vascular endothelial growth factor (vegf) and its receptors in human neuroblastoma. Eur. J. Cancer.

[54] Roy Choudhury S., Karmakar S., Banik N.L., Ray S.K. (2012). Targeting angiogenesis for controlling neuroblastoma. J. Oncol..

[55] Restin T., Kajdi M.E., Schlapfer M., Roth Z., Z’graggen B.R., Booy C., Dumrese C., Beck-Schimmer B. (2017). Sevoflurane protects rat brain endothelial barrier structure and function after hypoxia-reoxygenation injury. PLoS ONE.

[56] Fonseca A.C., Moreira P.I., Oliveira C.R., Cardoso S.M., Pinton P., Pereira C.F. (2015). Amyloid-beta disrupts calcium and redox homeostasis in brain endothelial cells. Mol. Neurobiol..

[57] Rosenstein J.M., Krum J.M., Ruhrberg C. (2010). VEGF in the nervous system. Organogenesis.

[58] Park D.S., Obeidat A., Giovanni A., Greene L.A. (2000). Cell cycle regulators in neuronal death evoked by excitotoxic stress: Implications for neurodegeneration and its treatment. Neurobiol. Aging.

[59] Bonda D.J., Lee H.P., Kudo W., Zhu X., Smith M.A., Lee H.G. (2010). Pathological implications of cell cycle re-entry in Alzheimer disease. Expert Rev. Mol. Med..

[60] Moh C., Kubiak J.Z., Bajic V.P., Zhu X., Smith M.A., Lee H.G. (2011). Cell cycle deregulation in the neurons of Alzheimer’s disease. Results Probl. Cell Differ..

[61] Dela Paz N.G., Walshe T.E., Leach L.L., Saint-Geniez M., D’Amore P.A. (2012). Role of shear-stress-induced vegf expression in endothelial cell survival. J. Cell Sci..

[62] Lutgendorf S.K., Cole S., Costanzo E., Bradley S., Coffin J., Jabbari S., Rainwater K., Ritchie J.M., Yang M., Sood A.K. (2003). Stress-related mediators stimulate vascular endothelial growth factor secretion by two ovarian cancer cell lines. Clin. Cancer Res..

[63] Boelens W.C. (2014). Cell biological roles of alphab-crystallin. Prog. Biophys. Mol. Biol..

[64] Bhat S.P., Gangalum R.K. (2011). Secretion of alphab-crystallin via exosomes: New clues to the function of human retinal pigment epithelium. Commun. Integr. Biol..

[65] Sreekumar P.G., Kannan R., Kitamura M., Spee C., Barron E., Ryan S.J., Hinton D.R. (2010). αB crystallin is apically secreted within exosomes by polarized human retinal pigment epithelium and provides neuroprotection to adjacent cells. PLoS ONE.

[66] Fu W., Vukojevic V., Patel A., Soudy R., MacTavish D., Westaway D., Kaur K., Goncharuk V., Jhamandas J. (2017). Role of microglial amylin receptors in mediating beta amyloid (abeta)-induced inflammation. J. Neuroinflammation.

[67] Isaacs A.M., Senn D.B., Yuan M., Shine J.P., Yankner B.A. (2006). Acceleration of amyloid beta-peptide aggregation by physiological concentrations of calcium. J. Biol. Chem..

[68] Naldi M., Fiori J., Pistolozzi M., Drake A.F., Bertucci C., Wu R., Mlynarczyk K., Filipek S., De Simone A., Andrisano V. (2012). Amyloid beta-peptide 25–35 self-assembly and its inhibition: A model undecapeptide system to gain atomistic and secondary structure details of the Alzheimer’s disease process and treatment. ACS Chem. Neurosci..

[69] LeVine H. (1993). Thioflavine t interaction with synthetic Alzheimer’s disease beta-amyloid peptides: Detection of amyloid aggregation in solution. Protein Sci..

[70] Fresta C.G., Chakraborty A., Wijesinghe M.B., Amorini A.M., Lazzarino G., Lazzarino G., Tavazzi B., Lunte S.M., Caraci F., Dhar P. (2018). Non-toxic engineered carbon nanodiamond concentrations induce oxidative/nitrosative stress, imbalance of energy metabolism, and mitochondrial dysfunction in microglial and alveolar basal epithelial cells. Cell Death Dis..

[71] Caruso G., Fresta C.G., Martinez-Becerra F., Antonio L., Johnson R.T., de Campos R.P.S., Siegel J.M., Wijesinghe M.B., Lazzarino G., Lunte S.M. (2017). Carnosine modulates nitric oxide in stimulated murine raw 264.7 macrophages. Mol. Cell Biochem..

[72] Fresta C.G., Hogard M.L., Caruso G., Melo Costa E.E., Lazzarino G., Lunte S.M. (2017). Monitoring carnosine uptake by raw 264.7 macrophage cells using microchip electrophoresis with fluorescence detection. Anal. Methods.

[73] Lopalco G., Lucherini O.M., Vitale A., Talarico R., Lopalco A., Galeazzi M., Lapadula G., Cantarini L., Iannone F. (2015). Putative role of serum amyloid-a and proinflammatory cytokines as biomarkers for behcet’s disease. Medicine.

[74] Zhu H., Xue X., Wang E., Wallack M., Na H., Hooker J.M., Kowall N., Tao Q., Stein T.D., Wolozin B. (2017). Amylin receptor ligands reduce the pathological cascade of Alzheimer’s disease. Neuropharmacology.

[75] Fu W., Patel A., Kimura R., Soudy R., Jhamandas J.H. (2017). Amylin receptor: A potential therapeutic target for Alzheimer’s disease. Trends Mol. Med..

