# Supplementary material for: Sub-Toxic Human Amylin Fragment Concentrations Promote the Survival and Proliferation of SH-SY5Y Cells via the Release of VEGF and HspB5 from Endothelial RBE4 Cells
Source: Int J Mol Sci. 2018 Nov 20;19(11):3659. doi: 10.3390/ijms19113659 (PMC6274958; doi:10.3390/ijms19113659)
Supplement: Supplementary file 1 [file ijms-19-03659-s001.pdf]

Table S1. RBE4 cells death induced by increasing concentrations of hA17-29.

CELL TREATMENT	LDH release (% of controls)
hA17–29 1 μM	102.33
	(2.08)
hA17–29 3 μM	106.71
	(1.26)
hA17–29 5 μM	125.83[a]
	(3.06)
hA17–29 10 μM	133.59[a]
	(4.32)

Lactate dehydrogenase (LDH) released by RBE4 cells following the treatment with increasing concentrations (1, 3, 5, and 10 μM for 48 h). LDH release (a measure of cell death) was measured in the cell culture medium using the Lactate Dehydrogenase Activity Assay Kit according to the provider's instructions. The change in absorbance of NADH, during reduction of lactate to pyruvate, was followed spectrophotometrically using using a microplate reader (LabSystems-Multiskan Ascent 354 Microplate Reader, San Diego, CA, USA). Data are the mean of four independent experiments (an average of four readings was considered for each sample) and are expressed as the percent variation with respect to the LDH release recorded in untreated (control) cultures. Standard deviations are in parenthesis. [a] Significantly different from untreated cells, $p < 0.01$.

Table S2. Effect of anti-Flk1 antibody pre-treatment (2 μg/mL - 2 h) on SH-SY5Y cells.

CELL TREATMENT	Absorbance at 569 nm (%) in absence of anti-Flk1	Absorbance at 569 nm (%) in presence of anti-Flk1	Difference
CM-RBE4	125.50	104.30[a]	-21.20
	(3.72)	(2.43)	
CM-RBE4-Aβ25–35	144.45	113.69[a]	-30.76
	(5.38)	(5.73)	
Aβ25–35 + CM-RBE4	110.85	102.11	-8.74
	(6.18)	(4.11)	
Aβ25–35 + CM-RBE4-Aβ25–35	120.90	109.23	-11.67
	(7.19)	(4.98)	

The [3-(4,5-dimethylthiazol-2-yl)-2,5-diphenyltetrazolium bromide] MTT assay was carried out by measuring absorbance at 569 nm as described in Figure 2. The conditioned medium (CM) was diluted into fresh media to a final concentration of 50%. Data are the mean of five independent experiments (an average of four readings was considered for each sample) and are expressed as the percent variation with respect to the absorbance detected in untreated control cultures. CM was diluted into fresh media, with a final concentration of 50%. The concentration of Aβ25–35 is 20 μM. Standard deviations are in parenthesis. Difference = Absorbance at 569 nm (%) in absence of anti-Flk1 – Absorbance at 569 nm (%) in presence of anti-Flk1. [a] Significantly different from corresponding treatment with no anti-Flk1, $p < 0.001$.

Table S3. SH-SY5Y cells death induced by the treatment for 48 h with a high concentration of Aβ25–35 and effect of conditioned media.

CELL TREATMENT	LDH release (% of controls) in absence of anti-Flk1	LDH release (% of controls) in presence of anti-Flk1
Aβ25–35	126.69[a] (3.35)	//
CM-RBE4	99.52 (3.20)	101.13 (5.01)
CM-RBE4-Aβ25–35	100.93 (2.72)	101.87 (4.11)
Aβ25–35 + CM-RBE4	116.20[b] (2.30)	118.80 (5.06)
Aβ25–35 + CM-RBE4-Aβ25–35	111.72[b] (3.10)	115.12 (4.73)

CM was diluted into fresh media to a final concentration of 50%. The concentration of Aβ25–35 was 20 μM. Cell death was determined using the LDH assay as described in Table S1. Data are the mean of four independent experiments (an average of four readings was considered for each sample) and are expressed as the percent variation with respect to the LDH release recorded in untreated (control) cultures. Standard deviations are reported in parentheses. [a] Significantly different from untreated cells, $p < 0.001$; [b] significantly different from Aβ25–35, $p < 0.01$.

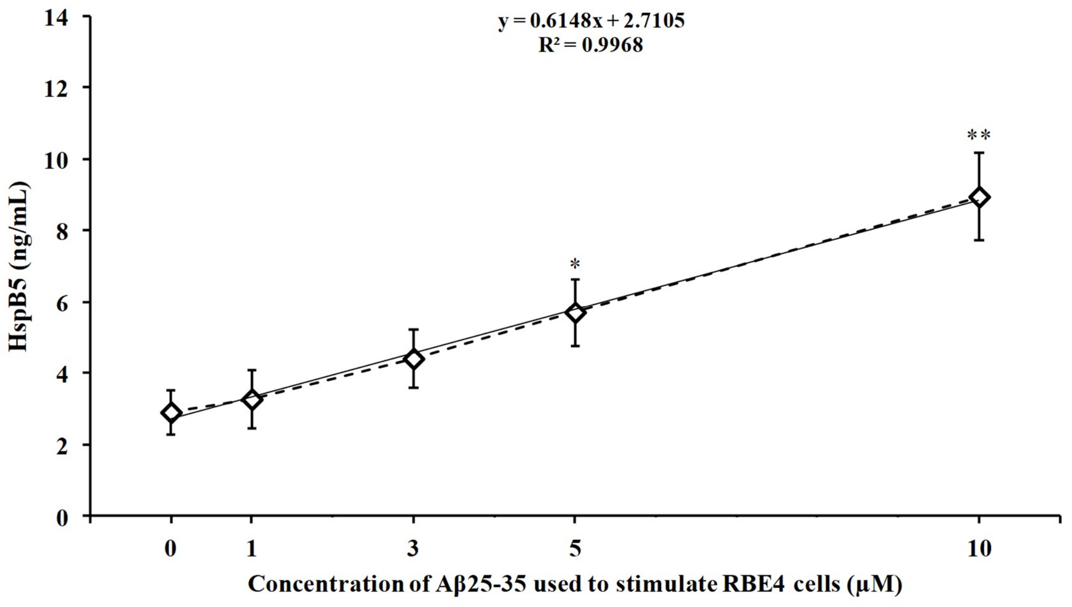

Figure S1. Effect of Aβ25–35 treatment on heat shock protein B5 (HspB5) secretion by RBE4 endothelial cells. RBE4 cells were stimulated for 48 h with different concentrations (1, 3, 5, and 10 μM) of freshly prepared Aβ25–35 peptide fragment. After 48 h, the medium was harvested and the amount of secreted HspB5 was quantified by ELISA as described in Figure 3. Data are the mean of three independent experiments (an average of four readings was considered for each sample). The dotted line is the trend of the experimental points (rhombus); the solid line is the best fitting straight line. * Significantly different from untreated (0 μM Aβ25–35), p < 0.05; * significantly different from untreated, p < 0.001.

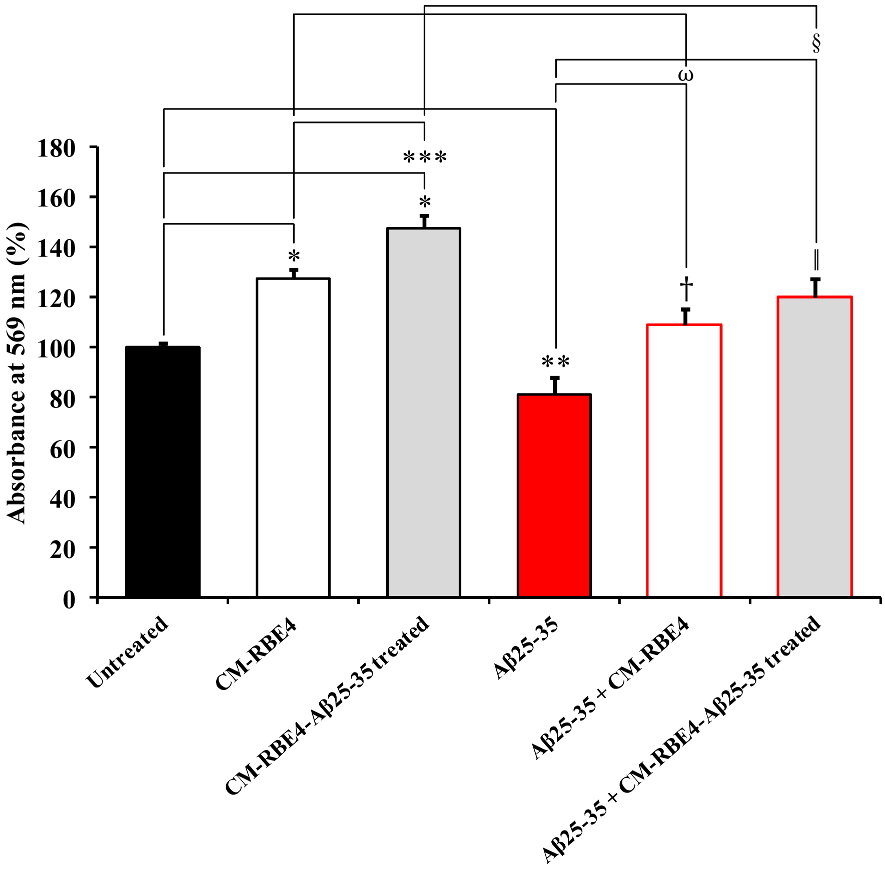

Figure S2. Conditioned medium (CM) derived from RBE4 cells counteracts SH-SY5Y Aβ25–35-induced toxicity. The MTT assay was carried out by measuring absorbance at 569 nm as described in Figure 2. Columns 2 and 3 show the effects on SH-SY5Y cells of the incubation for 48 h with CM obtained from untreated RBE4 (CM-RBE4) or RBE4 challenged with a sub-toxic (3 μM) concentration of Aβ25–35 (CM-RBE4-Aβ25–35). Column 4 clearly demonstrates the toxic effects due to the treatment with a high Aβ25–35 concentration (20 μM). Columns 5 and 6 depict the protective effects of both types of CM in counteracting, in SH-SY5Y, the decrease in absorbance induced by a toxic concentration (20 μM) of Aβ25–35. CM was diluted into fresh media, with a final concentration of 50%. Data are the mean of five independent experiments (an average of four readings was considered for each sample) and are expressed as the percent variation with respect to the absorbance at 569 nm detected in control cultures. Standard deviations are represented by vertical bars. * Significantly different from untreated cells, $p < 0.001$; ** significantly different from untreated cells, $p < 0.05$; *** significantly different from CM-RBE4, $p < 0.01$; † significantly different from Aβ25–35, $p < 0.01$; ‖ significantly different from Aβ25–35, $p < 0.001$; ω significantly different from corresponding treatment with no hA17-29, $p < 0.01$; § significantly different from corresponding treatment with no hA17-29, $p < 0.001$.
